# Analysing the Initial Bacterial Adhesion to Evaluate the Performance of Antifouling Surfaces

**DOI:** 10.3390/antibiotics9070421

**Published:** 2020-07-17

**Authors:** Patrícia Alves, Joana Maria Moreira, João Mário Miranda, Filipe José Mergulhão

**Affiliations:** 1 LEPABE—Laboratory for Process Engineering, Environment, Biotechnology and Energy, Faculty of Engineering, University of Porto, 4200-465 Porto, Portugal; up201510029@fe.up.pt (P.A.); joanarm@fe.up.pt (J.M.M.); 2 CEFT—Transport Phenomena Research Center, Faculty of Engineering, University of Porto, 4200-465 Porto, Portugal

**Keywords:** bacterial adhesion, blocking effect, hydrodynamics, parallel plate flow cell

## Abstract

The aim of this work was to study the initial events of *Escherichia coli* adhesion to polydimethylsiloxane, which is critical for the development of antifouling surfaces. A parallel plate flow cell was used to perform the initial adhesion experiments under controlled hydrodynamic conditions (shear rates ranging between 8 and 100/s), mimicking biomedical scenarios. Initial adhesion studies capture more accurately the cell-surface interactions as in later stages, incoming cells may interact with the surface but also with already adhered cells. Adhesion rates were calculated and results shown that after some time (between 5 and 9 min), these rates decreased (by 55% on average), from the initial values for all tested conditions. The common explanation for this decrease is the occurrence of hydrodynamic blocking, where the area behind each adhered cell is screened from incoming cells. This was investigated using a pair correlation map from which two-dimensional histograms showing the density probability function were constructed. The results highlighted a lower density probability (below 4.0 × 10^−4^) of the presence of cells around a given cell under different shear rates irrespectively of the radial direction. A shadowing area behind the already adhered cells was not observed, indicating that hydrodynamic blocking was not occurring and therefore it could not be the cause for the decreases in cell adhesion rates. Afterward, cell transport rates from the bulk solution to the surface were estimated using the Smoluchowski-Levich approximation and values in the range of 80–170 cells/cm^2^.s were obtained. The drag forces that adhered cells have to withstand were also estimated and values in the range of 3–50 × 10^−14^ N were determined. Although mass transport increases with the flow rate, drag forces also increase and the relative importance of these factors may change in different conditions. This work demonstrates that adjustment of operational parameters in initial adhesion experiments may be required to avoid hydrodynamic blocking, in order to obtain reliable data about cell-surface interactions that can be used in the development of more efficient antifouling surfaces.

## 1. Introduction

Bacterial adhesion to a surface triggers a series of events that may lead to biofilm formation and fouling of that surface. Biofilms are bacterial cell communities that are embedded in a self-produced and highly hydrated matrix of extracellular polymeric substances (EPS). Living in biofilms is the most common state for bacteria in natural environments [1], and biofilms can be composed of single or multiple species that interact with each other [2]. Biofilms can cause deterioration of industrial equipment, food spoilage and disease [3,4], but they are also used in wastewater treatment systems and have been investigated for the production of valuable molecules, including recombinant proteins [5,6].

The first step in biofilm formation is the surface adsorption of molecules from the surrounding medium. This originates a conditioning film that can affect subsequent bacterial adhesion [7]. Initially, bacterial adhesion is reversible, but then the adhered organisms start to produce EPS and to anchor themselves irreversibly, leading to the development of the biofilm structure. Then, biofilms mature and release bacteria, often leading to serious bacterial transmission issues [1]. Microorganisms that adhere first have a pivotal role in linking the biofilm to the surface and their retention is crucial to maintain the biofilm on that surface when it is challenged by shear forces [8]. Thus, a better understanding of the initial adhesion process may provide clues to the development of antifouling surfaces.

One of the most promising strategies to prevent or delay biofilm formation on a given surface is to use a coating. In both medical and industrial settings, different types of antifouling coatings have been developed to prevent adhesion, which operate by contact killing or that release biocidal agents [9]. Release systems may promote the development of different types of resistance, and contact killing surfaces may originate a layer of dead cells and cellular debris that can serve as anchoring points for subsequent cell adhesion [7]. In that case, newly adhered cells can be protected from the biocidal agent by the layer formed by dead cells and debris. Anti-adhesion systems are, therefore, an attractive way of preventing or delaying biofilm formation [10]. The development of anti-adhesion coatings is an intense area of research, but these coatings have to be tested in environmental conditions that mimic their application scenario. Since medical and industrial biofilms often develop in areas where significant fluid motion exists, fluid displacement systems have been used to study these initial bacterial–surface interactions [11]. One of the most commonly used platforms for adhesion studies is the parallel plate flow cell (PPFC), which enables real-time monitoring of bacterial adhesion when the system is mounted on a microscope stage coupled to an image acquisition system [11].

A typical initial adhesion experiment performed in a PPFC with continuous monitoring of the number of adhered cells generates a pattern where a linear trajectory can be identified first, followed by a decrease in slope where adhesion seems to be leveling off [8]. It has been proposed that during the linear phase, cells arriving at the surface interact solely with the surface and that the rate at which organisms adhere in this phase is truly representative of the affinity of that organism to that surface [8]. It is also believed that at later stages, when the surface is partially covered, an arriving cell will interact with the surface but also with other adhered cells and that the observed adhesion rates level off due to hydrodynamic blocking [8]. As a consequence, these second adhesion rates would not be truly representative of the interaction between a single cell and the surface, as data would reflect contributions from different interactions that are hard to separate [8].

**Figure 1:**
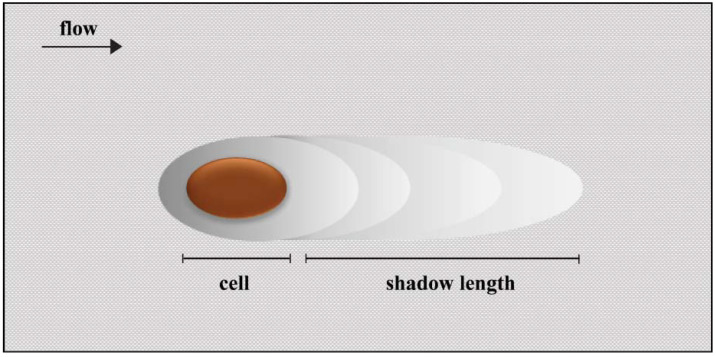
The hydrodynamic blocking effect. The area blocked by an adhered cell where further cell adhesion is prohibited is represented by the shadow.

It has been demonstrated that hydrodynamic blocking can reduce the adhesion of cells by screening the surface behind already adhered cells [12]. If the surface is entirely free of cells, an incoming cell can freely attach as long as it can withstand the shear forces. However, when cells start to attach irreversibly, an incoming cell can no longer attach immediately behind an already adhered cell because that area is effectively screened. This creates a shadow where cell adhesion is prohibited (Figure 1). During colloidal particle deposition, it has been shown that the area blocked by one particle can represent 8 to 675 times the cross-sectional area of that particle [13,14]. In general, blocking is more likely to occur at high surface coverages, but local flow effects can introduce anisotropy in cell adhesion [12].

A detailed analysis of the hydrodynamic blocking has been presented in several works by Adamczyk and co-workers [15,16,17], but this approach is not straightforward to many researchers, and this may explain why a blocking analysis is absent from many studies dealing with adhesion under flow conditions. In a study by van Loenhout et al. [12], the hydrodynamic blocking effect on particle adsorption was assessed by performing experiments in laminar flow and Monte Carlo simulations to evaluate the effect of the hydrodynamic shadow on particle distribution. The spatial distribution and anisotropy were analysed through a two-dimensional (2D) map of a pair correlation function that was obtained from image analysis. This enabled the observation of the exclusion zone originating from the hydrodynamic blocking. The position of the adhered particles could be calculated and revealed the density of particles adhering around a given particle when compared to the overall density.

The arrival of cells to the surface is dictated by mass transport, and in flow systems, this transport is achieved by convection and diffusion. Although solutions for the convective–diffusion equation can be obtained by complicated mathematical procedures, there are approximate solutions such as the Smoluchowski-Levich (SL) approximation that assumes that all microorganisms sufficiently close to a surface will adhere irreversibly [8]. On the other hand, adhered cells have to withstand hydrodynamic forces that may cause cell detachment, and therefore the adhesion rates observed in initial adhesion experiments are a balance between all these effects. It has also been shown that initial adhesion experiments can produce valuable data for the development of antifouling surfaces [10,18], and therefore the correct interpretation of that data is critical.

In this study, we have monitored in real-time the initial adhesion of *Escherichia coli* to a polydimethylsiloxane (PDMS) coating. *E. coli* was chosen as a model organism due to its relevance in both clinical and industrial settings, and PDMS is a very versatile polymer commonly used in both scenarios [19]. An interpretation of the events unfolding during initial adhesion is provided, showcasing the relative importance of cell transport to the surface, hydrodynamic blocking, and detachment forces.

## 2. Results and Discussion

Bacterial adhesion experiments were performed at different flow rates yielding a range of shear rates between 8 and 100/s, similar to relevant biomedical scenarios (Table S1) [20,21].

**Figure 2:**
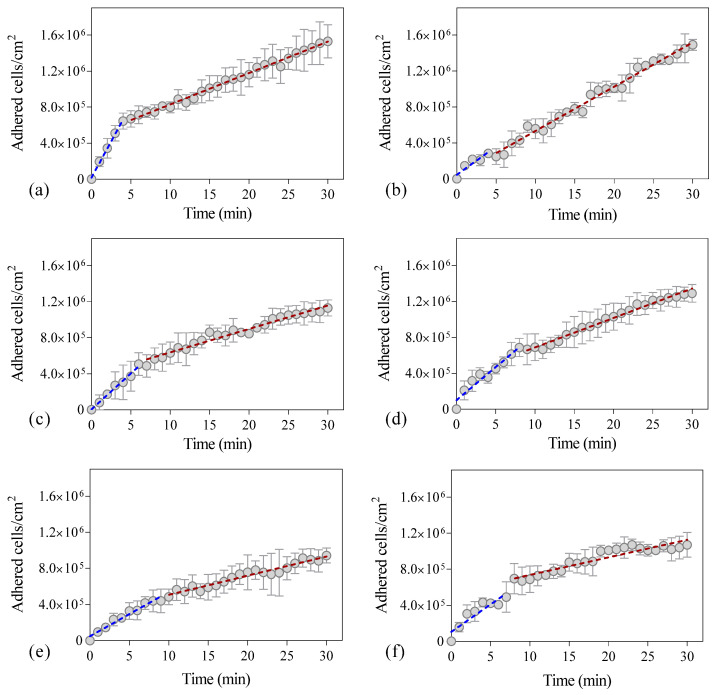
Number of *Escherichia coli* cells adhered to polydimethylsiloxane (PDMS) as a function of time. The dashed line indicates the best fit to a linear function for the initial adhesion rate (blue) and final adhesion rate (red) of the experiments. The adhesion assays were performed during 30 min at different flow rates: (**a**) 1 mL/s, (**b**) 2 mL/s, (**c**) 4 mL/s (**d**) 6 mL/s, (**e**) 8 mL/s, and (**f**) 10 mL/s corresponding to shear rates of 7.5/s, 15.0/s, 33.7/s, 51.6/s, 80.3/s, and 100.8/s, respectively, in a parallel plate flow cell (PPFC). Error bars indicate the standard deviation from three independent experiments.

**Figure 3:**
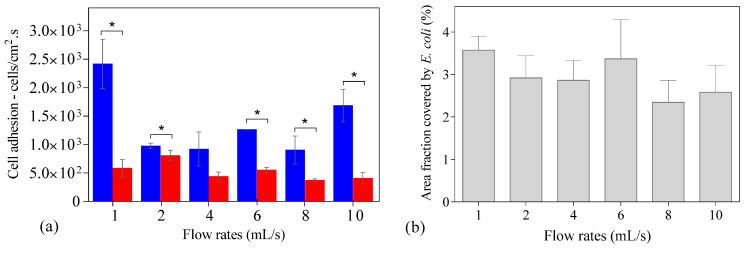
(**a**) Initial adhesion rates determined by linear regression of the first experimental points (blue bar) and for the remaining points (red bar) at different flow rates (1 to 10 mL/s). Statistical significance between the adhesion rates and the different flow rates was evaluated by one-way analysis of variance (one-way ANOVA, Tukey’s post-hoc test) (*p* < 0.05); (**b**) Area fraction covered by *Escherichia coli* cells at the end of adhesion assay (30 min) on polydimethylsiloxane (PDMS) at different flow rates (1 to 10 mL/s).

Figure 2 shows the number of cells adhered to the PDMS surface during the experimental time at different flow rates. In all tested conditions, an initial adhesion rate was determined by linear regression of the first experimental points (Figure 2, blue dashed line), and it was observed that after some time (between 5 and 9 min), the experimental points did not fit this initial regression. Thus, a second linear regression was made with the remaining points (Figure 2, red dashed line) so that a second adhesion rate could be determined. It was found that this second adhesion rate was lower (on average 55%) than the value determined with the first regression (in blue) (Figure 3a), but the most significant reduction was observed for the lower and higher flow rates (on average 76%; *p* < 0.05, Figure 3a). The flow rate of 2 mL/s presents the lowest reduction in the adhesion rate with a decrease of 20% (Figure 3a). Additionally, the adhesion rates were statistically different when comparing the results for the lower and the higher flow rates (1 and 2 mL/s with 8 and 10 mL/s, respectively) (*p* < 0.05).

It has been hypothesized that the main cause for this reduction in adhesion rate could be due to hydrodynamic blocking as arriving cells would interact with either already adhered cells or with the free surface [8]. Since the blocking effect is commonly evaluated as a function of the surface area coverage [12,22], this value was determined for the time where the slope decreased at each flow rate. Values between 0.8 to 1.8% were obtained, indicating low surface coverage at that time.

In order to assess if hydrodynamic blocking was occurring, we have performed an image analysis for each flow rate at the end of the adhesion assay (30 min), where surface coverage is at its highest value. It is precisely in those situations that blocking would be most likely to occur in our assays [12,22]. The image analysis revealed that the final surface coverage was similar for all tested flow rates (Figure 3b) and was below 4%.

**Figure 4:**
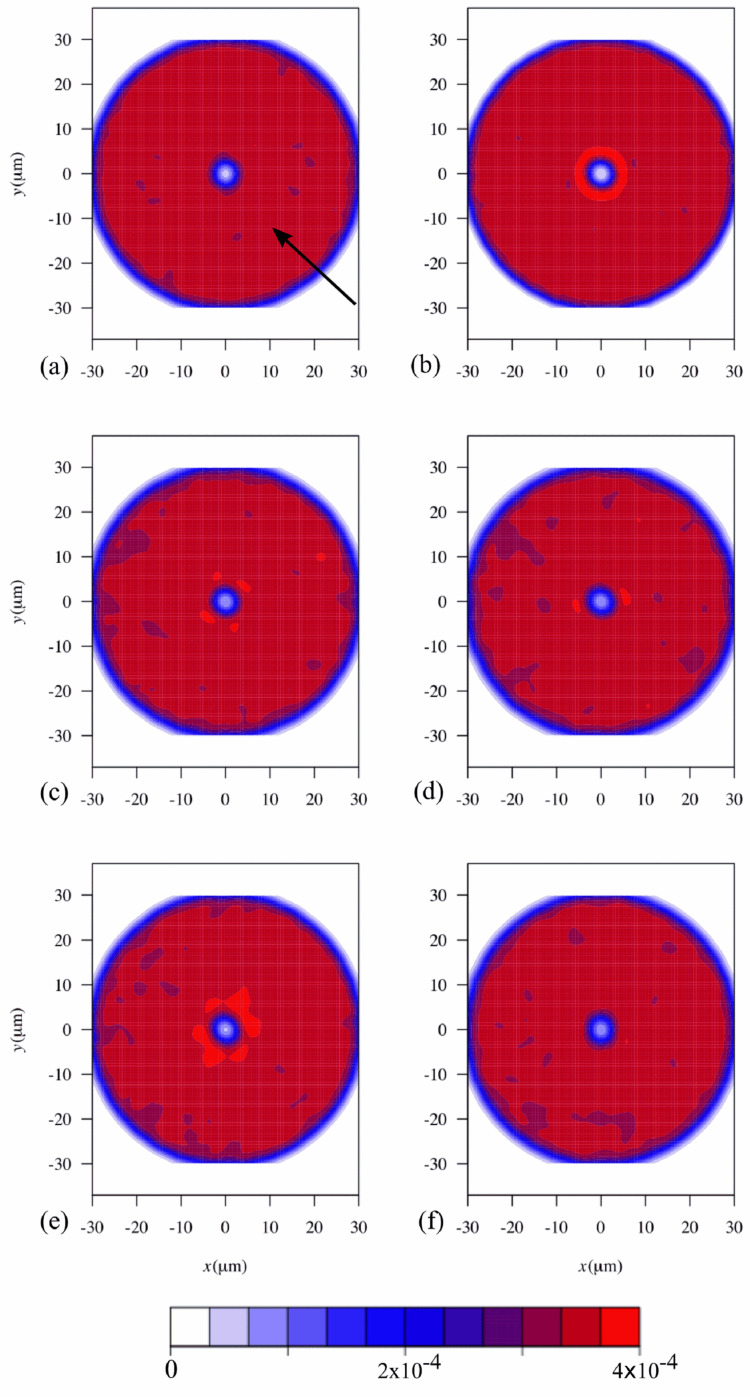
2D histograms representing the probability density function of the presence of a cell around a given cell (reference cell is in the center). Results for different flow rates are shown: (**a**) 1 mL/s, (**b**) 2 mL/s, (**c**) 4 mL/s (**d**) 6 mL/s, (**e**) 8 mL/s, and (**f**) 10 mL/s corresponding to shear rates of 7.5/s, 15.0/s, 33.7/s, 51.6/s, 80.3/s, and 100.8/s, respectively, in a PPFC. The number of independent cells measured for each flow rate was (**a**) 1.53 × 10^6^ ± 1.84 × 10^5^ cells/cm^2^; (**b**) 1.49 × 10^6^ ± 6.14 × 10^4^ cells/cm^2^; (**c**) 1.13 × 10^6^ ± 8.87 × 10^4^ cells/cm^2^; (**d**) 1.29 × 10^6^ ± 9.84 × 10^4^ cells/cm^2^; (**e**) 9.41 × 10^5^ ± 8.25 × 10^4^ cells/cm^2^ and (**f**) 1.07 × 10^6^ ± 1.37 × 10^5^ cells/cm^2^. The direction of the flow in all histograms is depicted by an arrow located on panel (a).

Afterward, a pair correlation map was used to establish if significant areas were blocked by already adhered cells. Then, 2D histograms were created, showing the probability density function of the presence of cells around a given cell (Figure 4). The central cell was excluded from the calculations, which explains the low probability in the center of the image. Cells at a distance larger than 50 pixels (30.5 µm) were also excluded from the analysis as it has been shown that blocking is more effective at distances shorter than the cut-off value used in this work [23]. If hydrodynamic blocking was occurring, the pair correlation map should show a low probability of cell adhesion along the flow direction, as demonstrated in previous studies [12]. However, in our experiments, the pair correlation maps are symmetrical, as the density probability of the presence of a cell around a given cell was uniform (Figure 4) and had no directional bias. This demonstrates that hydrodynamic blocking was not occurring during our experiments, not at the end of the assay and surely not at the time point where the adhesion rates decreased.

In a previous study, it was shown that the size of the blocked area is a function of the dimensionless Péclet number [24]. In the present work, the Péclet number was below 0.4, which is much lower than the scenarios simulated in that study (2 to 100). Indeed, the size of the blocked area was shown to increase at higher shear rates and large particle sizes [12], and this may also explain why a shadow area was not observed in our work. Additionally, the low surface coverage may also have prevented the occurrence of blocking as it has been reported that even at surface coverages of about 10%, the blocking effect may not be significant [22].

**Figure 5:**
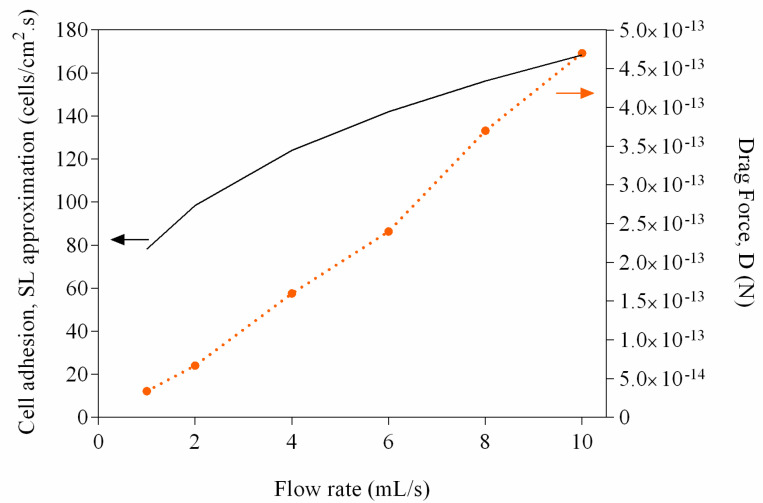
Variation of the drag force (D) and values obtained with the Smoluchowski-Levich (SL) approximation as a function of the flow rate (1 to 10 mL/s). The full line represents the predicted adhesion rate using the SL approximation and the dashed line represents the drag force.

Since hydrodynamic blocking was not occurring, other factors may explain the reduction in adhesion rates that we have observed (Figure 2). Higher flow velocities increase the number of contacts between planktonic cells and the surface, but the increased shear forces may also prevent adhesion or promote detachment [25,26]. In order to ascertain the effect of flow rate variation on the transport of cells from the bulk solution to the surface, the SL approximation was used [8]. Figure 5 shows that as the flow rate increases, mass transport is favored (values in the range of 80–170 cells/cm^2^·s were obtained), and since the SL approximation considers that all cells in close proximity to the surface will adhere, higher adhesion rates are expected at higher flow rates. However, as the flow rate increases, the wall shear stress also increases, and therefore higher drag forces are expected (values in the range of 3–50 × 10^−14^ N were determined—Figure 5). It has been shown that sufficiently high shear stresses cause adhering bacteria to slide and roll over a surface, which may lead to detachment [27].

It is known that the strength of adhesion can depend on the history of the contact between a bacterium and a surface and that factors like the residence time and the shear applied during adhesion are strong modulators [28,29]. Additionally, these forces are strongly depending on chemistry [30] and mechanical properties of the surface [31]. It is likely that for each experimental condition tested, the relative importance of mass transport and detachment changes, and this may induce variations in the observed cell adhesion. In any case, since blocking is not occurring, the experimental values obtained at this second stage are also a reflection of the interaction between single cells and the surface.

## 3. Materials and Methods

### 3.1. Bacteria and Culture Conditions

*E. coli* JM109(DE3) from Promega (USA) was selected for this study because it has been used in previous works from our group for the evaluation of initial adhesion in antifouling surfaces [10,18,32,33] and because it was shown to have similar biofilm formation behavior to different clinical isolates, including *E. coli* CECT 434 [21]. The inoculum was prepared as previously described [34]. Briefly, 500 µL of a glycerol stock (kept at −80 °C) was added to a total volume of 0.2 L of the inoculation medium composed by 5.5 g/L glucose (Chem-Lab nv, Zedelgem, Belgium), 2.5 g/L peptone (Oxoid, Basingstoke, Hampshire, England), 1.25 g/L yeast extract in phosphate buffer (1.88 g/L KH_2_PO_4_ and 2.60 g/L Na_2_HPO_4_; Chem-Lab nv, Zedelgem, Belgium) at pH 7.0. The culture was incubated overnight at 37 °C, with orbital agitation (160 rpm in a shaker: IKA KS 130 basic, Staufen, Germany). Subsequently, this culture was centrifuged (at 3202 *g* for 10 min at 25 °C) to harvest the cells, and these were washed twice with 0.05 M of citrate buffer (composed by citric acid, Scharlau, Barcelona, Spain; pH 5.0) to remove any traces of the culture medium [35]. Cells were again harvested by centrifugation and resuspended in citrate buffer by vortexing in order to reach an optical density of 0.1 (OD_610 nm_). A calibration curve was used to determine the cell density (7.6 × 10^7^ cells/mL). This suspension was used to perform adhesion experiments.

### 3.2. Surface Preparation and Experimental Setup

Adhesion experiments were performed in a PPFC, as described by Moreira et al. [20]. The PPFC used in the present work has a rectangular cross-section of 0.8 × 1.6 cm and a length of 25.42 cm. Briefly, glass slides (7.6 × 2.6 × 0.1 cm, VWR, Carnaxide, Portugal) were washed with a 0.5% detergent solution (Sonasol Pril, Henkel Ibérica SA, Barcelona, Spain) for 30 min and then the detergent was rinsed with distilled water. Subsequently, the surfaces were immersed in 3% sodium hypochlorite for 30 min. Finally, the surfaces were rinsed with distilled water and prepared for coating. PDMS (Sylgard 184 Part A, Dow Corning; viscosity = 1.1 cm^2^/s; specific density = 1.03, Midland, MI, USA) was prepared by performing the following steps: i) the curing agent (Sylgard 184 Part B, Dow Corning, Midland, MI, USA) was added to the PDMS at a 1:10 ratio; ii) the mixture was placed in the vacuum chamber in which the pump was turned on and off periodically thus changing the pressure and collapsing air bubbles that may have formed. Subsequently, the mixture was used to coat glass slides by spin-coating (Spin150 PolosTM, Caribbean, Netherlands) at 4000 rpm for 60 s in order to obtain a thickness of 10 µm. The PPFC was mounted in a microscope (Nikon Eclipse LV100, Tokyo, Japan).

### 3.3. Adhesion Experiments

The bacterial suspension was introduced in the flow cell for 30 min at flow rates of 1, 2, 4, 6, 8, and 10 mL/s. The flow rates were adjusted with a valve. Adhesion was followed by brightfield microscopy, and three trials were performed for each flow rate. Images were taken at 60 s intervals to enable a more accurate adhesion analysis. Obtained images were processed using ImageJ (version 1.38e) software [36] and for each flow rate tested, the number of adhered cells per unit area was determined as a function of time (Code 1—supplementary material). Initial adhesion rates were obtained by linear regression analysis of initial points. After some time, from 5 to 9 min, the adhesion rate decreased, and the remaining points were subjected to a second linear regression. The difference between the first and second slopes was analysed. Standard deviations on the triplicate sets were calculated for all analysed parameters. Graph production and statistical analysis were performed using GraphPad Prism 6.01 (La Jolla, USA). The differences between the slopes as well as the variation of the adhesion rates with the shear conditions were evaluated using one-way analysis of variance (one-way ANOVA, Tukey’s post-hoc test). All statistical analysis used a 95% confidence limit, so that *p* values equal to or greater than 0.05 were not considered statistically significant.

The percentage area fraction covered by *E. coli* cells was determined for different flow rates (1 to 10 mL/s). For this, ImageJ macro scripts were created to convert the images to an 8-bit greyscale file format and thresholds were applied to determine the occupied area (Code 2—supplementary material).

### 3.4. Blocking Analysis

After each 30 min trial, 5 images were taken in different regions of the surface to obtain a large set of adhered cells. Using ImageJ, the images were inverted, and the maxima detected (Figure S1). Each maximum corresponds to a cell. The respective coordinates were obtained and a pair correlation map was constructed [12]. Results are presented in the form of a 2D density probability function. The probability density function can be used to calculate, by integration, the probability of a cell being found around another cell at a region defined by ∆*x* and ∆*y*, where *x* and *y* are the coordinates centered in the reference cell. The probability density function was calculated using an R language script (see R scripts—supplementary materials). To find the probability density function, a threshold *R_max_* was defined. Pairs at a distance larger than *R_max_* were not considered to construct the probability density function.

### 3.5. Mass Transfer and Drag Force

The theoretical mass transport was estimated though the *SL* equation (approximate solution) [8] for each flow rate:

(1) SL=0.538D∞CbRb(Pe h0x)13,

where $D_{\infty }$ is the diffusion coefficient (approximately 4.0 × 10^−13^ m^2^/s for *E. coli* [37]), *C_b_* is the bacterial concentration (7.6 × 10^13^ cell/m^3^), *h*_0_ is the height of the rectangular channel (0.08 m) and *x* is the distance for which an average velocity variation below 15% was determined (m) as detailed in Moreira, et al. [20]. *R_b_* corresponds to the microbial radius (4.5 × 10^−7^ m) assuming that *E. coli* has a cylindrical shape estimated accordingly to the equation [15]:

(2) $$R_{b}=\frac{1}{\ln\left(\frac{L}{b}\right)-0.11}\left(\frac{L}{2}\right),$$

where *L* and *b* are the length and the diameter of the *E. coli* used in this study, respectively.

The *SL* equation also includes the Péclet number (*Pe*) which represents the ratio between convective and diffusional mass transport, given for the parallel plate configuration as:

(3) $$Pe=\frac{3v_{av}{R_{b}}^{3}}{2{\left(\frac{h_{0}}{2}\right)}^{2}D_{\infty }},$$

where $v_{av}$ is the average flow velocity (m/s) determined in Moreira et al. [20].

It is assumed that gravity effects and hydrodynamic lift are negligible compared to the drag force [38], which was estimated by the following equation [38]:

(4) D=32.0τwRb2+O(Rec),

where *τ_w_* corresponds to the wall shear stress.

The flow within the near-wall region can be characterized using the local Reynolds number (Re*_c_*), based on the shear rate, ϒ, [38], as follows:

(5) Rec=ρϒRb2/µ,

where ρ, is the density (993.37 kg/m^3^), and µ is the dynamic viscosity of the fluid (0.000694 kg/m.s). The values for *τ_w_* and ϒ were obtained by Computational Fluid Dynamics as described in [20] and are listed in Table S1 (Supplementary material).

Re*_c_* is always lower than 1, as $R_{b}\ll h_{0}$, and so the inertial effects are negligible in the near-wall region and terms of higher order, O(Rec), in Equation (4) are negligible [38].

## 4. Conclusions

Bacterial adhesion studies are important not only because they provide an understanding of the early stages of biofilm formation but also because they may provide clues for the development of more efficient antifouling surfaces. These studies should be performed in conditions that mimic the real-life scenario not only regarding the surfaces and bacteria under evaluation but also in defined hydrodynamic conditions prevailing on that scenario. When real-time monitoring of initial adhesion is performed, a decrease in the initial adhesion rates is often observed along the experimental time. This decrease is often associated with hydrodynamic blocking, which can occur at significant surface coverage values. For low surface coverage situations, this decrease is most likely caused by cell detachment, which occurs when the force exerted on a single bacterium overcomes the adhesion force between the cell and that surface. Initial adhesion experiments should, therefore, be conducted so that low surface coverage values are obtained (by adapting the test conditions, namely the assay time), and the absence of blocking should be verified so that reliable results can be obtained. This enables the performance evaluation of different coatings so that more efficient antifouling surfaces can be developed.

## Supplementary Materials

The following are available online at https://www.mdpi.com/2079-6382/9/7/421/s1, Figure S1: Steps of the method. From left to right: initial image, inverted image and maxima; R scripts—Relative positions script and Histogram script; Code 1—Cell adhesion analysis; Code 2—Surface coverage; Table S1: Shear rate and wall shear stress at the different flow rates tested (determined by Computational Fluid Dynamics) and examples of biomedical scenarios where these shear rates can be found.
