# Supplementary material for: Analysing the Initial Bacterial Adhesion to Evaluate the Performance of Antifouling Surfaces
Source: Antibiotics (Basel). 2020 Jul 17;9(7):421. doi: 10.3390/antibiotics9070421 (PMC7400106; doi:10.3390/antibiotics9070421)
Supplement: Supplementary file 1 [file antibiotics-09-00421-s001.pdf]

antibiotics

Supplementary Material

Patrícia Alves [1], Joana Maria Moreira [1], João Mário Miranda [2,*] and Filipe José Mergulhão [1,*]

[1] LEPABE - Laboratory for Process Engineering, Environment, Biotechnology and Energy, Faculty of Engineering, University of Porto, 4200-465 Porto, Portugal; up201510029@fe.up.pt (P.A.); joanarm@fe.up.pt (Joana.M.M.); filipem@fe.up.pt (F.J.M.)

[2] CEFT – Transport Phenomena Research Center, Faculty of Engineering, University of Porto, 4200-465 Porto, Portugal; jmiranda@fe.up.pt

* Correspondence: jmiranda@fe.up.pt (João.M.M.); filipem@fe.up.pt (F.J.M.); Tel.: (+351) 220414847 (João.M.M.); (+351) 225081668 (F.J.M.)

Received: 8 June 2020; Accepted: 15 July 2020; Published: date

Image processing and extraction of cell coordinates

After each 30 min trial, 5 images were taken in different regions of the surface to obtain a large set of adhered cells. Using ImageJ, the images were inverted, and the maxima detected (Figure S1). Each maximum corresponds to a cell. The respective coordinates were obtained and a pair correlation map was constructed [1]. Results were presented in the form of a 2D density probability function. The probability density function was calculated using two R language scripts. The file with the cell coordinates (input file) and the R language scripts are presented in the following sections.

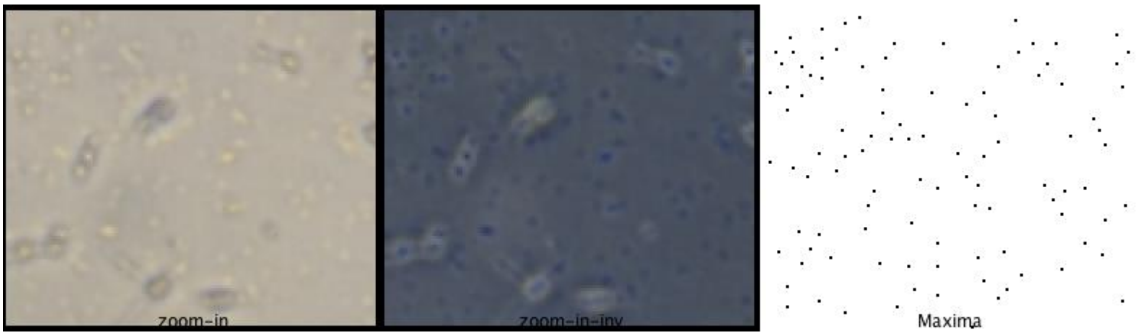

Figure S1. Steps of the method. From left to right: initial image, inverted image and maxima.

Input files

The input file for the R scripts that generate the 2D correlations maps has the following format (only the first 6 lines of the file are shown):

	X	Y
1	389	71
2	561	533
3	1191	539
4	417	63
5	1017	461

----

antibiotics

MDPI

R scripts

Two R language scripts were used to generate 2D correlations plot, a script that calculates the relative position in relation to the reference cell from the coordinates of the cells and a script that generates the histogram.

Relative positions script

```
      library(plotrix)

      rm(list=ls(all=TRUE))

# base path
      dir0="/Users/jmiranda/Desktop/results/"
#     coordinate files are here
      dirSub="t/"

#     relative coordinates directory (output of this script will be
saved here)
      dir_res_sub="t-res/"
      dir_res=paste(dir0,dir_res_sub,sep="")

      dir=paste(dir0,dirSub,sep="")
      text_t=character()

      ficheiros_nome=list.files(dir,pattern="*.txt",full.names     =
FALSE)
      ficheiro=paste(dir,ficheiros_nome[1],sep="/")

# cut off radius in pixels
      radius=50.0
      dados=read.table(ficheiro,header=TRUE,blank.lines.skip=FALSE)

for (temp in ficheiros_nome)
      {
# processes all available files

      aux=numeric()
      aux2=numeric()
      xx=numeric()
      yy=numeric()

      ficheiro=paste(dir,temp,sep="/")

      dados=read.table(ficheiro,header=TRUE,blank.lines.skip=FALSE)

      ficheiro_base=strsplit(temp,"\\.")[[1]]
      ficheiro_res0=paste(ficheiro_base[1],"res",sep="_")
      ficheiro_res0=paste(ficheiro_res0,"txt",sep=".")
      ficheiro_res=paste(dir_res,ficheiro_res0,sep="")

      xx=dados$X
      yy=dados$Y

      n=length(yy)

      xmax=max(xx)
      ymax=max(yy)

      for(i in (1:n))
            {
            if(xx[i]>radius  &  yy[i]>radius  &  xx[i]<(xmax-radius)  &
yy[i]<(ymax-radius))
```

antibiotics

MDPI

```
        {
        for(j in (1:n))
        {
        if(j!=i)
        {

        xp0=xx[i]
        yp0=yy[i]
        xp=xx[j]
        yp=yy[j]

        dist=sqrt((xp-xp0)^2+(yp-yp0)^2)

        if(dist<radius )
        {
        aux=c(aux,xp-xp0)
        aux2=c(aux2,yp-yp0)
        }
        }}}}

    x <- data.frame(a = aux, b = aux2)
    write.table(x, file = ficheiro_res, sep = ",", col.names = NA,
           qmethod = "double")
    }
```

Histogram script

```
    library(plotrix)

    library(MASS)

    require(grDevices) # for colours

    rm(list=ls(all=TRUE))


#   base path
    dir0="/Users/jmiranda/Desktop/results/"
#   directory with the coordinates files

    dirSub="t-res/"



#   full path

    dir=paste(dir0,dirSub,sep="")




    ficheiros_nome=list.files(dir,pattern="*.txt",full.names     =
FALSE)


    temp=ficheiros_nome[1]

    count=numeric()
```

antibiotics

MDPI

```
    for (temp in ficheiros_nome)
        {

        dir2=paste(dir0,"t-tif/",sep="")
        nome=paste(dir2,temp,sep="")
        nome=paste(nome,".tiff",sep="")

        tiff(filename = nome, , res = 300, width = 1600, height = 1600)

        ficheiro=paste(dir,temp,sep="/")

    dados=read.table(ficheiro,sep=",",header=FALSE,blank.lines.skip=FALSE,skip=1)

#       0.61 is the conversion factor from pixels to micrometers
        x=dados$V2*0.61
        y=-dados$V3*0.61

#       maximum and minimum values
        valmax=4e-4
        valmin=0

        df <- data.frame(x,y)
        k <- kde2d(x, y,n=50)
        clo=""
        rgb.palette <- colorRampPalette(c("white", "blue", "red"),space = "rgb")
        clo=rgb.palette(12)
        a=seq(valmin,valmax,(valmax-valmin)/12)

      filled.contour(k, col= clo,levels = a , zlim=range(valmin,valmax),asp = 1,plot.title=title(main = temp,
    xlab = expression(paste(italic(x),"(",symbol(m),"m)")), ylab = expression(paste(italic(y),"(",symbol(m),"m)")) )  )

        dev.off()
        }
```

CODE 1 (Cell adhesion analysis):

```
rename("Original");
run("8-bit");
run("Subtract Background...", "rolling=20 light stack");
run("Set Scale...", "distance=1000 known=110 unit=um");
setAutoThreshold("Default");
setOption("BlackBackground", false);
run("Convert to Mask", "method=Default background=Light calculate");
run("Analyze Particles...", "size=1-Infinity show=Outlines display
exclude clear summarize stack");
```

CODE 2 (Surface coverage):

```
run("8-bit");
run("Invert""stack");
run("Subtract Background..." "rolling=10 light stack");
run("Color Balance...");
setAutoThreshold("Default dark");
run("Threshold...");
call("ij.plugin.frame.ThresholdAdjuster.setMode"    "Red");
setAutoThreshold("Default");
run("NaN Background"    "stack");
run("NaN Background"    "stack");
run("Set Measurements..."    "area area_fraction limit redirect=None
decimal=3");
run("Analyze Particles..." "show=Outlines display exclude clear
summarize stack");
```

Table S1. Shear rate and wall shear stress at the different flow rates tested (determined by Computational Fluid Dynamics) and examples of biomedical scenarios where these shear rates can be found. Adapted from Alves, et al. [2].

Flow Rate (mL/s)	Wall Shear Stress (τ_w, Pa)	Shear Rate (ϒ, 1/s)	Biomedical Scenarios
1	0.0052	7.5	Fluid flow in the oral cavity
2	0.0102	15.0	Urinary flow in a catheter
4	0.0237	33.7	Flow in femoral artery
6	0.0362	51.6	Blood flow in venous vessels
8	0.0566	80.3	Flow in ascending aorta
10	0.0719	100.8	Flow in ascending aorta

References

1. van Loenhout, M. T.; Kooij, E. S.; Wormeester, H.; Poelsema, B., Hydrodynamic flow induced anisotropy in colloid adsorption. *Colloids and surfaces A: Physicochemical and engineering aspects* **2009**, 342, (1-3), 46-52. doi: 10.1016/j.colsurfa.2009.03.058

2. Alves, P.; Nir, S.; Reches, M.; Mergulhão, F., The effects of fluid composition and shear conditions on bacterial adhesion to an antifouling peptide-coated surface. *MRS Communications* **2018,** 8, (3), 938-946. doi: 10.1557/mrc.2018.160
